# Sigma1 inhibitor suppression of adaptive immune resistance mechanisms mediated by cancer cell derived extracellular vesicles

**DOI:** 10.1080/15384047.2025.2455722

**Published:** 2025-01-26

**Authors:** Paola A. Castagnino, Derick A. Haas, Luca Musante, Nathalia A. Tancler, Bach V. Tran, Rhonda Kean, Alexandra R. Steck, Luis A. Martinez, Elahe A. Mostaghel, D. Craig Hooper, Felix J. Kim

**Affiliations:** a Department of Pharmacology, Physiology, and Cancer Biology, Thomas Jefferson University, Philadelphia, PA, USA; b Sidney Kimmel Comprehensive Cancer Center at Jefferson, Philadelphia, PA, USA; c University of Pennsylvania School of Veterinary Medicine, Philadelphia, PA, USA; d Geriatric Research, Education and Clinical Center, U.S. Department of Veterans Affairs Puget Sound Health Care System, Seattle, WA, USA

**Keywords:** Sigma1/*SIGMAR1*, extracellular vesicle (EV), programmed death ligand 1 (PD-L1), EV-associated PD-L1 (evPD-L1), Interferon-gamma (ifn-γ), adaptive immune resistance (AIR), tumor microenvironment (TME), T cell, antitumor immune response, endoplasmic reticulum (ER), indoleamine 2, 3-dioxygenase 1 (IDO)

## Abstract

Adaptive immune resistance in cancer describes the various mechanisms by which tumors adapt to evade anti-tumor immune responses. IFN-γ induction of programmed death-ligand 1 (PD-L1) was the first defined and validated adaptive immune resistance mechanism. The endoplasmic reticulum (ER) is central to adaptive immune resistance as immune modulatory secreted and integral membrane proteins are dependent on ER. Sigma1 is a unique ligand-regulated integral membrane scaffolding protein enriched in the ER of cancer cells. PD-L1 is an integral membrane glycoprotein that is translated into the ER and processed through the cellular secretory pathway. At the cell surface, PD-L1 is an immune checkpoint molecule that binds PD-1 on activated T-cells and blocks anti-tumor immunity. PD-L1 can also be incorporated into cancer cell-derived extracellular vesicles (EVs), and EV-associated PD-L1 can inactivate T-cells within the tumor microenvironment. Here, we demonstrate that a selective small molecule inhibitor of Sigma1 can block IFN-γ mediated adaptive immune resistance in part by altering the incorporation of PD-L1 into cancer cell-derived EVs. Sigma1 inhibition blocked post-translational maturation of PD-L1 downstream of IFN-γ/STAT1 signaling. Subsequently, EVs released in response to IFN-γ stimulation were significantly less potent suppressors of T-cell activation. These results suggest that by reducing tumor derived immune suppressive EVs, Sigma1 inhibition may promote antitumor immunity. Sigma1 modulation presents a novel approach to regulating the tumor immune microenvironment by altering the content and production of EVs. Altogether, these data support the notion that Sigma1 may play a role in adaptive immune resistance in the tumor microenvironment.

## Introduction

Programmed death-ligand 1 (PD-L1, also known as B7-H1 or CD274) is a type I integral membrane glycoprotein that can be induced to express on the surface of many cell types.^1^ Upon binding its cognate receptor, programmed death-1 (PD-1, also known as CD279), PD-L1 acts as an inhibitory checkpoint molecule and suppresses T cell activation and subsequent proliferation.^1^ PD-1/PD-L1 blockade of the host immune response normally serves to protect against autoimmunity and excessive tissue damage at the site of inflammation.^1^ However, many cancers co-opt this protective feature of the immune system to evade antitumor immunity.^2–5^ In the context of cancer, PD-L1 on the surface of cancer cells binds to PD-1 on tumor-infiltrating cytotoxic T lymphocytes (CTLs) and inactivates these important contributors to the host’s antitumor immune response.^1^ Immunomodulatory checkpoint inhibitor antibodies that block PD-L1/PD-1 interactions have exhibited remarkable clinical efficacy in subsets of patients with melanoma, non-small cell lung cancer, metastatic bladder cancer, and renal cell carcinoma.^6–9^ However, only a relatively low percentage of patients respond to anti-PD-1/PD-L1 monotherapy.^6,7,10^ Furthermore, in many patients who initially respond to immune checkpoint blockade such as anti-PD-1/PD-L1 therapy, tumors can adapt and become resistant to this immunotherapy thus limiting the duration and magnitude of their anti-tumor response.^11–13^ The mechanisms underlying resistance to checkpoint blockade are not well understood.^11–13^

Emerging data suggest that chronic upregulation of interferon-gamma (IFN-γ) response pathways is a key driver of acquired resistance.^11–13^ So-called adaptive immune resistance (AIR) in cancer describes the various mechanisms by which tumors adapt to evade anti-tumor immune responses. IFN-γ induction of PD-L1 was the first defined and therapeutically validated AIR mechanism.^14,15^ PD-L1 upregulation in response to IFN-γ is a key mechanism of AIR.^3–5,14,16^ IFN-γ is a pluripotent cytokine that plays a central role in the maintenance of immune system homeostasis^17^. It can promote anti-tumor immunity by inducing the proliferation of CTLs, such as CD8+ T cells and natural killer (NK) cells.^3–5,14,18^ IFN-γ plays a central role in coordinating and balancing innate and adaptive immunity by activating the immune responses to eliminate pathogens and tumors and preventing immune over-activation with associated, nonspecific tissue damage. The complex, context-dependent mechanisms that maintain this balance remain poorly defined.^12,13,17,18^

Mechanistic studies of PD-L1 mediated immune suppression in the TME have largely focused on the actions of PD-L1 expressed on the surface of cancer cells. However, PD-L1 is also incorporated into cancer cell-derived extracellular vesicles (EVs) that create a sort of immunosuppressive barrier to the tumor.^19,20^ EVs comprise heterogeneous populations of cellular membrane derived vesicles that vary in size, morphology, density, and content.^21,22^ EVs contain an array of proteins, lipids, and nucleotides, and the physical characteristics and biochemical composition of EVs determine their functions.^23–25^ There is a rapidly growing number of biological activities associated with EVs; however, much remains unknown regarding the underlying molecular mechanisms that govern EV biogenesis and regulation of EV content and their biological actions.^23–25^ EV-associated PD-L1 (evPD-L1) has been shown to suppress activated T cells both *in vitro* and *in vivo*, thereby decreasing both T cell proliferation and production of cytotoxic mediators.^19^ IFN-γ has been reported to increase PD-L1 incorporation into cancer cell derived EVs^19^ and IFN-γ enhanced PD-L1 expression by EVs has been reported to contribute to the immune suppressive tumor microenvironment (TME).^26–28^ Cancer patients are reported to have elevated levels of circulating evPD-L1, and these higher circulating evPD-L1 levels have been shown to correlate with a poor response to anti-PD-1 therapy.^19^

EVs are dependent on components of the secretory pathway, particularly the endoplasmic reticulum (ER).^23,24^ The ER is an elaborate and extensive network of membrane cisternae and tubules that occupies much of the cytoplasmic space.^29,30^ A rapidly growing body of data demonstrates that the interconnected and contiguous ER network forms membrane contact sites (MCSs) with many organelles, including the plasma membrane (PM) and endosomes.^31–33^ The ER directly interacts with endosomes and influences their trafficking, cargo sorting, and fission through MCSs.^31–34^ ER-endosome MCSs can directly regulate the sorting of PM proteins that are internalized into vesicles guided and modified through the endolysosomal sorting pathway, and these endosomes sort cargo during the routing and maturation process.^33,35^ The factors and mechanisms that govern the selective sorting of EV cargo are complex and remain poorly defined.^21,22,36^ The cellular factors that regulate the production and activities of EVs in AIR are also poorly understood.^26,27,37^

The ER is central to AIR as immune modulatory secreted and integral membrane proteins are dependent on ER. Sigma1 (gene name *SIGMAR1*; also known as sigma-1 receptor) is a unique pharmacologically responsive intracellular integral membrane scaffolding protein.^38,39^ Sigma1 is enriched in the secretory pathway, particularly the ER of most cells.^38,39^ Sigma1 itself has no known intrinsic signaling or enzymatic activity, rather it allosterically modulates the intracellular signaling and activities of its associated proteins.^38–44^ The multifunctionality of Sigma1 enables it to regulate lipid and protein homeostasis at multiple levels and it contributes to protein synthesis, processing, trafficking, assembly, and quality control in the secretory pathway of cells.^38–40,44^ We, and others, have shown that inhibition of Sigma1 disrupts ER protein and membrane dynamics through the secretory pathway.^44–46^

While it has previously been shown that Sigma1 inhibition can induce autophagic degradation of PD-L1, here, we hypothesized that pharmacological inhibition of Sigma1 could block aspects of IFN-γ mediated adaptive immune resistance through effects on PD-L1 expression and incorporation into immune modulatory EVs. We discovered that a selective small molecule inhibitor of Sigma1 blocked IFN-γ-mediated upregulation of PD-L1 at a post-translational level. Furthermore, Sigma1 inhibitor treatment decreased incorporation of PD-L1 into cancer cell derived EVs. The EVs produced by Sigma1 inhibitor-treated cancer cells interfered with IFN-γ-induced EV mediated T cell inactivation. Altogether, these data support the notion that Sigma1 could play a role in adaptive immune resistance in the TME through both intracellular and extracellular mechanisms.

## Materials and methods

### Chemicals

IPAG (1-(4-Iodophenyl)-3-(2-adamantyl) guanidine) was purchased from Tocris. Recombinant human Interferon-gamma (IFN-γ) was purchased from Gibco. Endoglycosidase H (Endo H) was purchased from New England Biolabs.

### Cell lines

PC3 (androgen receptor negative prostate adenocarcinoma), MDA-MB-231 (triple negative breast cancer), MDA-MB-436 (triple negative breast cancer), and HT-29 (colon cancer) cells were purchased from ATCC. WM164 (melanoma) cells were purchased from Rockland, Inc. Cells lines were authenticated by short tandem repeat profiling. All cancer cell lines were maintained in RPMI-1640 supplemented with 10% fetal bovine serum (Corning) at 37°C with 5% CO_2_.

### Immunoblotting and antibodies

Cell lysis, SDS-PAGE, and immunoblotting were performed as described previously.^44^ Immunoblotted proteins were revealed using Luminata Western HRP Substrate Chemiluminescence Kit (Millipore). The rabbit anti-PD-L1 (E1L3N XP, catalog # 13684), rabbit anti-STAT1 (D1K9Y, catalog # 14994), rabbit anti-phospho-STAT1 Y701 (58D6, catalog # 9167), rabbit anti-RCC1 (D15H6, catalog # 5134), rabbit anti-Calnexin (C5C9, catalog # 2679), rabbit anti-cleaved PARP (D64E10, catalog # 5625), rabbit anti-Sigma1 (D4J2E, catalog # 61994), and rabbit anti-HRS (D7T5N, catalog # 15087) antibodies were purchased from Cell Signaling Technology. The mouse anti-CD9 (C-4, catalog # sc -13,118), mouse anti-CD81 (B-11, catalog # sc -166,029) and mouse anti-GAPDH (6C5, catalog # sc -32,233) antibodies were purchased from Santa Cruz Biotechnology. The rabbit anti-CD63 antibody (catalog # ab68418) was purchased from Abcam. The rabbit anti-Na(+)/K(+) ATPase antibody (catalog # 14418–1-AP) was purchased from Proteintech. Where indicated, cell lysates were treated with endoglycosidase H (New England Biolabs) according to the manufacturer’s instructions before separation by SDS – PAGE.

### PD-L1 transcript analysis by quantitative reverse transcriptase PCR (qRT-pcr)

PD-L1 mRNA transcript levels were quantified essentially as previously described, with minor modifications.^40^ Briefly, cells were treated approximately 24 hours after seeding with 10 ng/mL IFN-γ for 16 hours in the presence or absence of 10 µmol/L of the Sigma1 inhibitor, IPAG. Total cellular RNA was extracted from cells using the RNeasy Kit (Qiagen) per the manufacturer’s protocol. Taq-Man primer probe sets were purchased from Life Technologies, and the genes and catalog numbers used for the qRT-PCR experiments are the following: PD-L1 (Hs00204257_m1) and GAPDH (Hs99999905-m1). The reactions were performed in triplicate using the Brilliant II qRT-PCR Master Mix 1-Step Kit (Agilent Technologies) following the manufacturer’s instructions. qRT-PCR was performed using the QuantStudio 12K Flex Real Time PCR System (Applied Biosystems). PD-L1 transcripts were normalized to GAPDH transcripts.

### Nuclear and cytosolic fractionation

Cells were seeded approximately 24 hours prior to treatment with 10 ng/mL IFN-γ for 16 hours in the presence or absence of 10 µmol/L of the Sigma1 inhibitor, IPAG. Cells were detached from the plate using 0.25% trypsin-EDTA (Corning), centrifuged at 500 × *g* for 5 minutes and then resuspended DPBS. 5 × 10^6^ cells were transferred to an Eppendorf tube and the nuclear and cytosolic fractions were isolated using NE-PER Nuclear and Cytoplasmic Extraction kit (Thermo Scientific) per the manufacturer’s instructions.

### Flow cytometry

Cells were seeded, and 24 hours later, treated with 10 ng/mL IFN-γ for 16 hours in the presence or absence of 10 µmol/L of the Sigma1 inhibitor, IPAG. Cells were detached from the plate using 2 mmol/L EDTA (Corning). Cells were washed in Dulbecco’s modified phosphate buffered saline (DPBS) (Corning) and aliquoted with 1 × 10^6^ cells per tube. Cells were then fixed in 4% methanol-free formaldehyde (Sigma-Aldrich) for 15 minutes at 37°C. Cells were washed twice in staining buffer (0.5% bovine serum albumin in PBS). Cells were stained with the rabbit anti-PD-L1 extracellular domain-specific (D8T4X) Alexa Fluor 488 (Cell Signaling Technology, catalog # #86744) at 1:100 for 60 minutes at 37°C. Cells were washed and resuspended in staining buffer. Fluorescence of each cell population was analyzed on the BD LSR II flow cytometer using FACS Diva Software (BD Biosciences) and reported as mean fluorescence intensity (MFI) relative to control.

### Biotinylation of cell surface proteins

Cells were seeded approximately 24 hours prior to treatment with 10 ng/mL IFN-γ for 16 hours in the presence or absence of 10 µmol/L of the Sigma1 inhibitor, IPAG. Cells were detached from the plate using a cell scraper and then washed in ice cold DPBS, pH 8.0 three times to remove any contaminating proteins or broken cells. Cells were resuspended in ice cold DPBS, pH 8.0 at a concentration of 25 × 10^6^ cells/mL, and freshly prepared 10 mm Sulfo-NHS-SS-Biotin (ThermoFisher) was added per the manufacturer’s instructions. Cells were incubated at room temperature for 30 minutes. Cells were washed once in ice cold 50 mm Tris, pH 8.0 to quench any non-reacted biotin reagent and then an additional three times with ice cold DPBS, pH 8.0.

### Isolation of extracellular vesicles (EVs)

Five million PC3 cells were plated in 15-cm dishes and allowed to grow for 48 hours in RPMI-1640 supplemented with 5% fetal bovine serum. Cells were then rinsed with DPBS to remove all cellular and bovine EVs. Serum-free medium was added to the cells containing 10 ng/mL IFN-γ in the presence or absence of the Sigma1 inhibitor, IPAG (10 µmol/L). Twenty-four hours later, the supernatant was collected from the cells and processed by filtration and differential centrifugation, described below. The cells were collected and counted using a Countess II automated cell counter (ThermoFisher) to determine vesicle production per cell. First, the supernatants were cleared of dead cells and cell debris by centrifuging at 2,000 RCF for 30 minutes at 4°C. Subsequently, EV isolation was performed by hydrostatic filtration dialysis (HFD) first followed by differential centrifugation. HFD was performed as described previously, with modifications.^47^ Cellulose ester (CE) dialysis membrane with molecular weight cutoff (MWCO) 1000 kDa width 16 mm, 0.79 mL/cm (Repligen # 131486) and/or width 31 mm 3.1 mL/cm (Repligen # 131492) were used to concentrate 300 mL and 1,200 mL of CM media respectively. The system setup was set according to.^48^ All the parts were abundantly sprayed with 70% (v/v) ethanol and let dry for at least 1 hour in a biological safety cabinet. The samples were first concentrated by gravity to a final volume of 10 mL; the filtration happened inside the biological safety cabinet. The concentrated CM (10 mL) was recovered from the dialysis by opening the clips on top of a 50 mL tube (we referred to it as HFDa^47^. Thereafter, the concentrated CM was transferred to a 70 mL capped polycarbonate bottle (Beckman Coulter, catalog # 355655) and centrifuged at 14,000 rpm for 35 minutes at 4°C using a 45 Ti fixed angle rotor (k factor 133 at max speed; Beckman Coulter) installed in an Optima L-90K Ultracentrifuge (Beckman-Coulter). The supernatant (SN) was poured into a clean polycarbonate bottle and centrifuged at 40,000 rpm for 35 minutes at 4°C using a 45 Ti fixed angle rotor. All the pellets obtained were resolubilized in 1 ml of DPBS filtered (DPBS^0.1μm^) with 0.1 μm syringe filter (Minisart® PES syringe filter, Sartorious) and transferred to a 1 mL thick-wall polycarbonate tube (Beckman Coulter, catalog # 362305). Tubes were centrifuged at 25,000 rpm for 35 minutes using a TLA-110 fixed angle rotor (k factor 13 at max speed; Beckman Coulter) installed in an Optima TLX-120 benchtop ultracentrifuge (Beckman-Coulter). Pellets were resolubilized in 200 μL DPBS^0.1μm^. This procedure is illustrated in Supplemental Figure 1.

**Figure 1.**
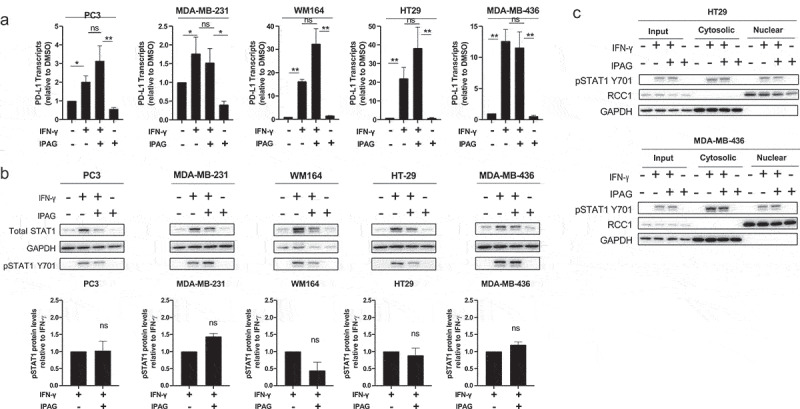
Pharmacological inhibition of Sigma1 does not prevent the formation of PD-L1 transcripts or STAT1 phosphorylation and translocation into the nucleus in response to IFN-γ. (a) qRT-pcr quantification of PD-L1 transcripts for MDA-MB-231, PC3, WM164, HT29, and MDA-MB-436 cell lines treated for 16 hours with DMSO, 10 ng/mL IFN-γ, 10 ng/mL IFN-γ and 10 µmol/L IPAG, or 10 µmol/L IPAG alone. Data represent mean values from three independent determinations and error bars represent SEM. *p < .05, **p < .01, ns = no significance. (b) Immunoblot of total STAT1 and phospho-STAT1 Y701 from whole-cell protein extracts of MDA-MB-231, PC3, WM164, HT29, and MDA-MB-436 cell lines treated for 16 hours with DMSO, 10 ng/mL IFN-γ, 10 ng/mL IFN-γ and 10 µmol/L IPAG, or 10 µmol/L IPAG. Immunoblots were quantified by densitometry for each cell line. Data are presented as fold induction over IFN-γ treated control samples and represent mean values from two independent determinations and error bars represent SD. (c) Isolation of nuclear and cytosolic fractions of HT29 and MDA-MB-436 cells treated for 16 hours with DMSO, 10 ng/mL IFN-γ, 10 ng/mL IFN-γ and 10 µmol/L IPAG, or 10 µmol/L IPAG showing that the translocation of pSTAT1 into the nucleus is not inhibited. Immunoblot showing phospho-STAT1 Y701, RCC1 (nuclear marker), and GAPDH (cytosolic marker).

### Nanoparticle tracking analysis (NTA)

NTA was performed using the ZetaView PMX-220 Twin (Particle Metrix) configured with 488 nm and 640 nm lasers with long wave-pass (LWP) cutoff filters (500 nm and 660 nm respectively) and a sensitive CMOS camera 640 × 480 pixels. Samples were diluted in 2 mL of 0.1 μm filtered (Minisart^Ⓡ^ high flow hydrophilic 0.1 μm syringe filter Sartorious) deionized water (DI 18 MΩ/cm) to obtain a particle concentration between 1 × 10^7^ and 1 × 10^8^ particles/mL (50–200 particles). The instrument was set to a sensitivity of 80, a shutter speed of 100, and a frame rate of 30 frames per second (fps). Each sample was measured at 11 different positions throughout the cell, with 1 cycle of reading at each position to have a minimum of 1000 traces. If the number of traces was below 1000 counts some additional sample was flushed inside the cell and the acquisition was repeated. Post-acquisition parameters were set to a minimum brightness of 20, a maximum size area of 1000 pixels, a minimum size area of 10 pixels, and a trace length of 15 frames. Automated cell quality control was checked using high quality deionized water (DI). Camera alignment and focus optimization were performed using polystyrene Nanosphere^TM^ 100 nm size standard beads (Thermo Scientific, catalog # 3100A). Data analysis was performed with ZetaView 8.05.14 software provided by the manufacturer. Automated reports of the particles recording across the 11 positions were manually checked, and any outlier position was removed to calculate particle concentration and distribution.

### Discontinuous density gradient separation of EV populations

The EV pellets obtained after the first round of centrifugation after HFD concentration were re-solubilized in 1,600 μL of iodixanol (Optiprep 60% (w/v) stock solution in water, SIGMA, catalog # D1556-250 ML) diluted with a 0.25 M sucrose/10 mm Tris/1 mm EDTA buffer solution at pH 7.4 for a final concentration of 30% (w/v) iodixanol and placed at the bottom of a 3.5 mL thick-wall polycarbonate tube (13 × 51 mm tube, Beckman Coulter, catalog # 349622) A discontinuous iodixanol gradient was generated by layering with a syringe and G21 needle successive 700 μL of 20%, and 700 μL of 10% (w/v) iodixanol in filtered with 0.1 μm syringe filter (Minisart® PES syringe filter, Sartorious) 0.25 M sucrose/10 mm Tris/1 mm EDTA buffer solutions layered on top of the discontinuous gradient. Tubes were spun at 53,700 rpm for 1 hour at 4°C using a SW55 Ti rotor (k factor at 48 at max speed; Beckman Coulter) installed in an Optima L-90K Ultracentrifuge (Beckman-Coulter). Ten 300 µL gradient fractions were collected by a syringe and G21 needle from top to bottom of the discontinuous density gradient and the densities of blank (no sample) discontinuous gradient were determined using an ABBE-3 L refractometer (Fisher Scientific). The fractions were then diluted to 1 mL using DPBS^0.1μm^, transferred to 1 mL thick-wall polycarbonate tube (Beckman Coulter, catalog # 362305), and centrifuged at 80,000 rpm for 60 minutes for all the density fractions obtained from the P20 pellets using a TLA-110 fixed angle rotor (k factor 13 at max speed; Beckman Coulter) installed in an Optima TLX-120 benchtop ultracentrifuge (Beckman-Coulter). Pellets were resolubilized for immunoblotting in RIPA buffer supplemented with a cocktail of protease and phosphate inhibitors.

### Cryogenic electron microscopy (Cryo-EM)

Of the isolated EV pellets suspended in DPBS, 3 µL were applied onto Quantifoil holey carbon grids and plunge frozen in liquid ethane using a Vitrobot Mark IV. Images were taken on a Titan Krios G3i equipped with a K3 Bioquantum.

### Immunofluorescent staining for Nano-fcm analysis

Mouse anti-human CD9 FITC-conjugated antibody (clone HI9a, Biolegend, catalog # 312104), mouse anti-human CD63 AF-647-conjugated antibody (clone H5C6, Biolegend, catalog # 353016), and mouse anti-human PD-L1 PE-conjugated antibody (clone B7-H1, Biolegend, catalog # 329705) were used for immunofluorescent staining of EVs. One hundred ng corresponding to 1 μL anti-CD9 and 0.5 μL of anti-CD63 were added to 5 μL of P20-EV sample with a particle concentration of approximately 5 × 10^10^ particles/ml. Two hundred ng corresponding to 1 μL of anti-human PD-L1 was added to 5 μL of P20-EV sample with a particle concentration of approximately 5 × 10^10^ particles/ml. The mixture was incubated at room temperature for 60 minutes. Buffer and EVs plus antibodies were diluted 100 times with DPBS filtered with 0.02 μm Whatman™ Anotop™ syringe filter (Cytiva, catalog # 6809–3002). The flow nano-analyzer nFCM (NanoFCM Co. Ltd) was used to measure particle concentration and size of particles following the manufacturer’s instructions. Briefly, two single photon-counting avalanche photodiodes (APDs) platform was used for the simultaneous detection of side scatter (SSC; SPCM-1 detector, bandpass filter 488/10) and fluorescence of individual particles (FITC, SPCM-2 detector, bandpass filter 525/40; AF-647, SPCM-3 detector, bandpass filter 670/30 and PE, SPCM-3 detector, bandpass filter 580/40). HPLC-Ultrapure water (SIGMA, catalog # 900682-4 L) served as the sheath fluid via gravity feed. The instrument was aligned and calibrated with 200 nm fluorescence reference beads for concentration and a cocktail of silica beads (68, 91, 113, and 151 nm) for size, respectively (NanoFCM Co. Ltd). Using the calibration curve, the flow rate and side scattering intensity were converted into corresponding particle number and size. Unstained samples were analyzed to determine the concentration and the size of the EVs. The particle number in the 1-minute acquisition was targeted to be in the 2000–12,000 events/min range recommended by the company.

### T cell activation assay

Human T cells were isolated from whole blood using Lymphoprep (Stemcell) according to the manufacturer’s protocol. Briefly, whole blood was diluted with an equal volume of PBS + 2% FBS, layered onto Lymphoprep, and centrifuged at 800 g for 25 minutes at room temperature. The mononuclear cell layer was collected, pooled, and washed with PBS + 2% FBS. Cells were resuspended at a concentration of 5 × 10^7^ per mL in PBS and subjected to magnetic separation using the EasySep direct human T cell isolation kit (Stemcell) according to the manufacturer’s protocol. T cells were either left unstimulated or activated with 25ul Immunocult Human CD3/CD28/CD2 (Stemcell) and incubated at 37C and 5% CO2 for 3 days.

### T cell co-culture with EVs and flow cytometry

T cells were collected, washed, and plated at 25,000 cells per well in 100 µl Immunocult-XF T cell expansion media. EVs (2.8 × 10^10^ particles/ml) were added at 10 µl per well in triplicate. Cultures were incubated for 4 hours at 37C and 5% CO2. Replicate wells were pooled and washed twice with FACS buffer. Cells were stained for 1 hour with antibodies specific for human CD4 (Miltenyi, 130-113-228) and CD8 (Miltenyi, 130-110-680). T cells were analyzed on a Guava 8 hT flow cytometer (EMD Millipore). Data analysis was performed using FlowJo software (Tree Stars Inc.).

### Trypan blue assay

Cells were detached from the plate using 0.25% trypsin-EDTA (Corning). The trypsin was washed away, and the cells were resuspended in DPBS (Corning). Cell suspensions were mixed in a 1:1 ratio with trypan blue, and the percent live cells were determined in quadruplicate using a Countess II automated cell counter (ThermoFisher).

### Statistical analysis

To determine the statistical significance of single comparisons, an unpaired Student’s t-test was used. To determine the statistical significance of multiple comparisons, a one-way ANOVA with Bonferroni posttest was performed using Prism software (GraphPad).

## Results

### Pharmacological inhibition of Sigma1 does not suppress PD-L1 transcript levels and does not block IFN-γ mediated STAT1 phosphorylation and translocation to the nucleus

IFN-γ canonically signals through the JAK/STAT1/3 pathway and mediates an array of transcriptional responses.^3–5,14,16,17,49,50^ After STAT1 is phosphorylated, it dimerizes and is translocated to the nucleus, where it binds to the promoters of interferon stimulated genes, including PD-L1.^3–5,14,16^ We asked whether pharmacological inhibition of Sigma1 suppresses PD-L1 by blocking the upstream JAK/STAT1/3 signaling cascade that drives transcription regulation of PD-L1 or whether the effects of Sigma1 modulation occur primarily downstream at a post-translation step. In this study, we used IPAG (1-(4-Iodophenyl)-3-(2-adamantyl)guanidine), a selective small molecule Sigma1 inhibitor.^39^ The Sigma1 specificity of IPAG has been demonstrated by several orthogonal studies in multiple publications (reviewed in^39^).

In this study, rather than focusing on a specific cancer type, we focused on cancer cell lines that either constitutively express high levels of PD-L1 or inducible lines that express low or undetectable basal levels of PD-L1 in the absence of IFN-γ. We chose MDA-MB-231 and PC3 cells to examine the effect of IPAG on cell lines with high levels of constitutive PD-L1 expression. PC3 cells were also chosen because of their ability to produce relatively large quantities of EVs with associated PD-L1.^20^ The low basal PD-L1 expressing WM164, HT29, and MDA-MB-436 cells were chosen to examine the effects of IPAG on cell lines with IFN-γ inducible PD-L1. Importantly, the lack of high basal PD-L1 expression in the latter group of cell lines permitted examination of the effects of IPAG on nascent PD-L1.

Consistent with previously published reports, IFN-γ induced PD-L1 transcripts in all of the cell lines tested here (Figure 1a).^16^ Co-treatment with IPAG did not block IFN-γ mediated upregulation of PD-L1 transcripts, and in the majority of cell lines, there were more PD-L1 transcripts, up to two-fold more, compared to IFN-γ treatment alone (Figure 1a). IFN-γ treatment resulted in increased STAT1 protein levels and increased phospho-STAT1 levels (Figure 1b). IPAG did not significantly reduce IFN-γ induced phospho-STAT1 levels (Figure 1b). IPAG also did not prevent phospho-STAT1 from translocating into the nucleus of the cells (Figure 1c), which is required for IFN-γ mediated transcriptional induction of PD-L1.^16^ Therefore, IPAG did not prevent IFN-γ mediated transcriptional upregulation of PD-L1.

While most publications have focused on the immunomodulatory actions of PD-L1, relatively little is known about the intrinsic cancer cell signaling activities of PD-L1. PD-L1 has a conserved cytoplasmic sequence that is important for apoptotic resistance to both type I and type II interferon cytotoxicity.^51^ Additionally, human cancer samples that acquire enhancing mutations within this region have been shown to have increased resistance to pro-apoptotic signaling of interferons.^52^ Thus, the ability of IPAG to prevent PD-L1 from progressing through the secretory pathway and reaching the cell surface would have a detrimental effect on cancer cell survival in the presence of sustained IFN-γ signaling. This might create a feedback loop wherein the cell increases the production of new PD-L1 to compensate for the loss of PD-L1 signaling and potentially explain why multiple cell lines show elevated PD-L1 transcript levels following combined treatment with IFN-γ and IPAG (Figure 1a).

### Pharmacological inhibition of Sigma1 blocks post-translational modification and maturation of IFN-γ-induced PD-L1

**Figure 2.**
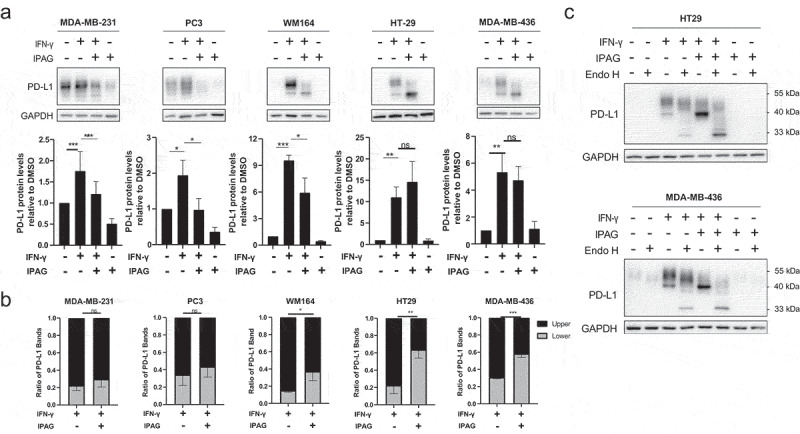
Pharmacological inhibition of Sigma1 blocks post-translational modification and maturation of IFN-γ-induced PD-L1. (a) Immunoblot of PD-L1 from whole-cell protein extracts of MDA-MB-231, PC3, WM164, HT29, and MDA-MB-436 cell lines treated for 16 hours with DMSO, 10 ng/mL IFN-γ, 10 ng/mL IFN-γ and 10 µmol/L IPAG, or 10 µmol/L IPAG. Immunoblots were quantified by densitometry for each cell line. Data are presented as fold induction over dmso-treated control samples and represent mean values from at least three independent determinations and error bars represent SEM. *p < .05, **p < .01, ***p < .001, ns = no significance. (b) Quantification of the upper molecular weight PD-L1 band ranging from 45–55 kDa versus the lower molecular weight PD-L1 band ranging from 35–45 kDa showing the accumulation of lower molecular weight PD-L1 when IFN-γ treatment is combined with IPAG. Data are presented as fold induction over IFN-γ treated samples and represent mean values from at least three independent determinations and error bars represent SEM. *p < .05, **p < .01, ***p < .001, ns = no significance. (c) Immunoblot of endoglycosidase H treated whole-cell protein extracts from HT29 and MDA-MB-436 cell lines treated for 16 hours with DMSO, 10 ng/mL IFN-γ, 10 ng/mL IFN-γ and 10 µmol/L IPAG, or 10 µmol/L IPAG showing that the accumulated PD-L1 band at 40 kDa in the combination treatment is incompletely glycosylated.

We previously demonstrated that Sigma1 physically associates with PD-L1 and triggers its lysosomal degradation.^40^ Here, we asked whether the Sigma1 inhibitor, IPAG, would prevent nascent PD-L1 from maturing and progressing through the secretory pathway in cell lines from multiple types of cancers. In all the cell lines tested, IFN-γ upregulated PD-L1 protein levels, consistent with the increased levels of PD-L1 mRNA transcripts (Figure 1a). When IPAG was combined with IFN-γ in cells that constitutively express high levels of PD-L1, PD-L1 protein levels decreased (as previously reported^40^) (Figure 2a). In the low basal expressing, IFN-γ inducible PD-L1 cell lines (WM164, HT29, and MDA-MB-436), PD-L1 protein was still produced in the presence of IPAG (Figure 2a), indicating that IPAG did not block PD-L1 translation. However, in the low basal expressing lines, the combination of IFN-γ and IPAG resulted in the accumulation of PD-L1 at a lower molecular weight band, at approximately 40 kDa, that was not seen with IFN-γ alone (Figure 2a). Quantification of the banding pattern (Figure 2b) confirmed that there was a higher percentage of the PD-L1 migrating at approximately 40 kDa in the IFN-γ and IPAG combination treatment condition compared to treatment with IFN-γ alone in the low basal PD-L1 expressing lines.

We previously found that Sigma1 is associated with nascent PD-L1,^40^ and others have reported that differential banding patterns of PD-L1 could be the result of differential N-linked glycosylation.^53^ Therefore, we asked whether the accumulated lower molecular weight PD-L1 band reflected inhibition of proper glycosylation. To answer this question, we treated cell lysates with endoglycosidase H (Endo H), which cleaves all immature, high-mannose N-linked glycans in the ER and cis Golgi before glycoproteins are processed by the enzyme Golgi alpha-mannosidase II in the medial Golgi, after which the glycans are no longer susceptible to cleavage by Endo H.^54^ We found that the ~ 40 kDa PD-L1 band that accumulated in the IFN-γ and IPAG combination treatment condition was Endo H sensitive (Figure 2c). As the lower molecular weight band did not migrate at 33 kDa (which would represent the non-glycosylated PD-L1), but rather ~ 40 kDa, it appears that IPAG did not prevent the initial attachment of the N-linked glycans, rather prevented its proper trimming and maturation. Thus, IPAG did not prevent the maturation of nascent PD-L1 by disrupting N-linked glycosylation. Rather, IPAG prevented the maturation of nascent PD-L1 by disrupting its proper trimming and maturation.

### Pharmacological inhibition of Sigma1 decreases cell surface PD-L1 levels

**Figure 3.**
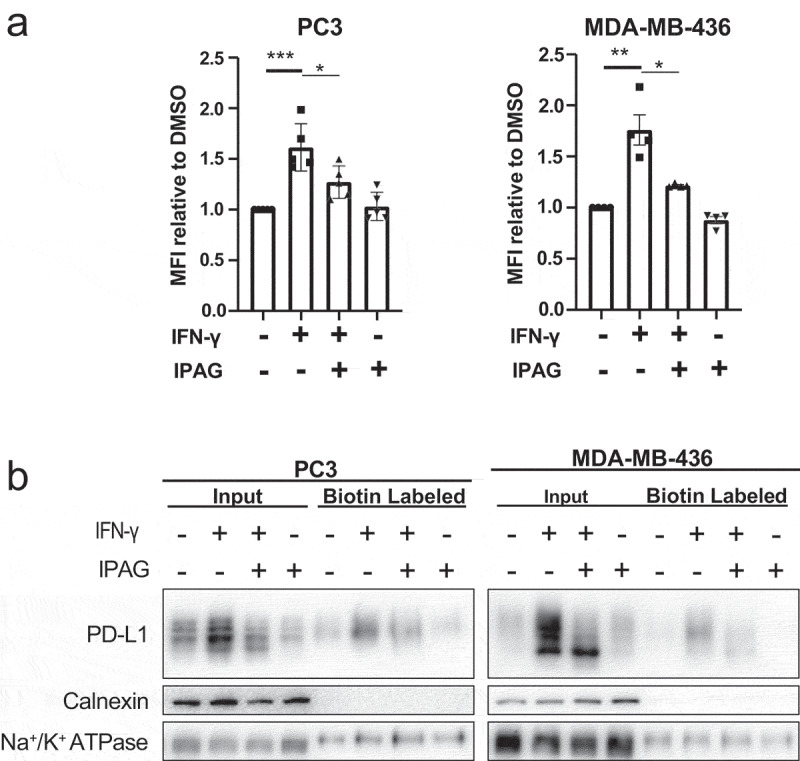
Pharmacological inhibition of Sigma1 suppresses IFN-γ induced PD-L1 cell surface expression. (a) Flow cytometric analysis of cell surface PD-L1 in PC3 and MDA-MB-436 cell lines treated for 16 hours with DMSO, 10 ng/mL IFN-γ, 10 ng/mL IFN-γ and 10 µmol/L IPAG, or 10 µmol/L IPAG from at least four independent determinations and error bars represent SEM. *p < .05, **p < .01, ***p < .001. (b) Cell surface biotinylation of PD-L1 MDA-MB-436 and PC3 cell lines showing that less PD-L1 is at the cell surface when IPAG is combined with IFN-γ treatment.

IFN-γ mediated upregulation of PD-L1 also increases PD-L1 cell surface expression.^16,53^ Here, we confirmed that IFN-γ induced cell surface PD-L1 levels in representative examples of high (PC3) and low basal PD-L1 expressing (MDA-MB-436) cell lines (Figure 3). By flow cytometry, we found that IPAG prevented constitutively expressed and IFN-γ induced PD-L1 expression at the surface of these cells (Figure 3a). We performed cell surface protein biotinylation studies to confirm this effect using an orthogonal method which has the added advantage of detecting distinct banding patterns and thus PD-L1 protein post-translational modifications (Figure 3b). In MDA-MB-436 cells, IFN-γ increased cell surface PD-L1 levels; however, in response to IFN-γ and IPAG combination, the lower ~ 40 kDa band produced was not biotinylated. This indicates that the Endo H sensitive band of PD-L1 was not present at the cell surface (Figure 3b). The cell compartment control calnexin (ER-membrane protein) was not biotinylated, while the Na^+^/K^+^-ATPase (cell surface protein) was biotinylated (Figure 3b). Of note, this further supports the concept that pharmacological inhibition of Sigma1 did not impact all cell surface and integral membrane proteins. Thus, IPAG selectively decreased IFN-γ mediated cell surface expression of PD-L1.

### Pharmacological inhibition of Sigma1 suppresses EV-associated PD-L1 (evPD-L1)

**Figure 4.**
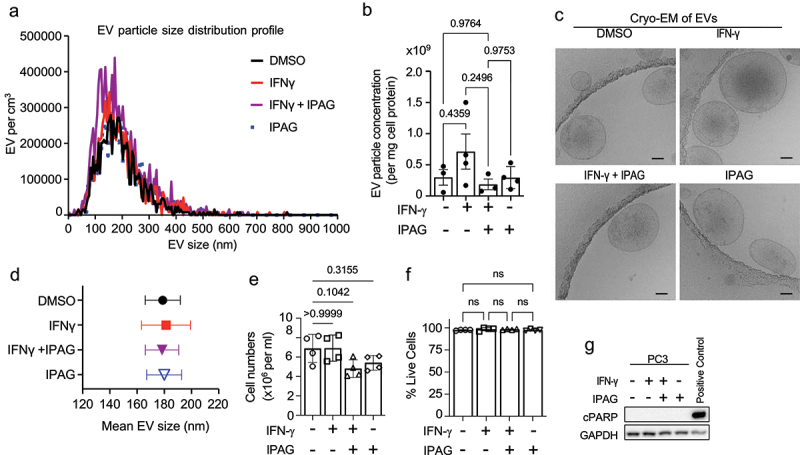
Isolated of extracellular vesicles (EVs) from prostate cancer cells treated with IFN-γ and Sigma1 inhibitor. PC3 cells were treated with DMSO, 10 ng/mL IFN-γ, 10 ng/mL IFN-γ + 10 µmol/L IPAG, or 10 µmol/L IPAG for 24 hours. (a) Representative zetaview particle tracking analysis of EV size distribution. (b) Relative number of EV particles (x10^9^) per milligram (mg) of cell protein measured by zetaview nanoparticle analysis. This was determined for *N* = 3 to 4 batches of EV preparations. P-values for each comparison to the control condition are indicated. Although more EVs appeared to be produced by IFN-γ treated PC3 cell, the difference did not reach significance in this study. (c) Cryogenic electron microscopy (Cryo-EM) images of the EVs from each treatment condition. Scale bar represents 50 nm. (d) Mean EV particle size in nanometers (nm). Error bars represent SEM. (e) Number of cells collected from each treatment cell culture condition. Note that decreased numbers of cells in IFN-γ and IFN-γ + IPAG conditions reflect decreased proliferation, but not cell death. *N* = 4 per condition. P-values for each comparison to the control condition are indicated. There was no significant difference. (f) The percentage of live cells from each culture condition determined by trypan blue exclusion assay. *N* = 4 per condition. Not significant (ns). (g) Immunoblot of cleaved poly(adp-ribose) polymerase (cPARP) as a marker of apoptosis. ShRNA knockdown of p97/VCP consistently induced apoptosis and was used as a positive control.

A growing body of evidence demonstrates that Sigma1 is a multifunctional chaperone or scaffolding protein that contributes to the maintenance of ER protein homeostasis in cancer cells and supports their increased utilization of the secretory pathway.^39^ EV production and its physical and biochemical characteristics are dependent on the secretory pathway.^23,24^ Therefore, we asked whether IPAG would alter the production and characteristics of EVs. Zetaview nanoparticle tracking analysis (NTA) showed no salient differences in the overall size profile of EVs produced by cells treated with vehicle (DMSO), IFN-γ, IFN-γ with IPAG, or IPAG alone (Figure 4a,b). Analysis of the EV pellets by cryogenic electron microscopy (Cryo-EM) confirmed the presence of vesicles mostly ranging in size from 50–200 nm (Figure 4c). Under these conditions, IPAG treatment also did not induce cell death or apoptosis, which we confirmed by trypan blue exclusion assay and the absence of cleaved PARP, respectively (Figure 4d,e).

**Figure 5.**
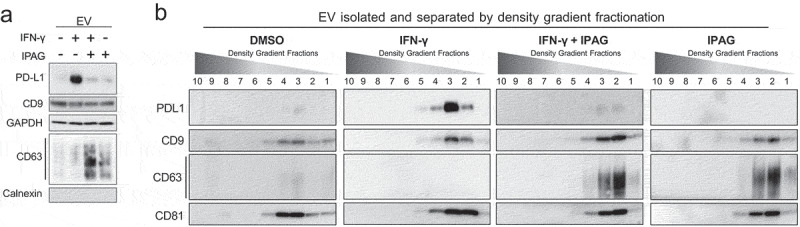
Pharmacological inhibition of Sigma1 prevents IFN-γ induced PD-L1 incorporation into EVs. PC3 cells were treated for 16 hours with DMSO, 10 ng/mL IFN-γ, 10 ng/mL IFN-γ and 10 µmol/L IPAG, or 10 µmol/L IPAG. (a) Immunoblot of EVs from PC3 cells. 1 × 10^9^ EVs were loaded per lane. (b) Immunoblots of EVs separated on an iodixanol density gradient showing PD-L1 colocalizing in fractions with canonical tetraspanin EV markers CD9, CD63, CD81.

Next, we asked whether pharmacological inhibition of Sigma1 would also decrease PD-L1 incorporation into EVs as well as decrease the number of EVs. To be packaged into EVs, PD-L1 must first be expressed on the cell surface.^20^ From there, PD-L1 can enter the endosomal sorting pathway where it can be packaged into EVs or recycled to the cell surface.^20^ Since IPAG prevented PD-L1 from reaching the cell surface, we hypothesized that it would inhibit PD-L1 incorporation into EVs. Others have reported that IFN-γ promotes PD-L1 incorporation into EVs.^19,20^ Here, we reproduced these results and found increased levels of PD-L1 in EVs produced by IFN-γ treated PC3 cells (Figure 5a,b). IPAG prevented IFN-γ-induced PD-L1 from being incorporated into the EVs (Figure 5a,b).

Interestingly, while IPAG prevented PD-L1 from being incorporated into EVs, it did not suppress other EV-associated proteins, including CD9, CD81, and CD63 (Figure 5a,b). Tetraspanins, including CD9 and CD63, are scaffolding proteins that are thought to mediate selective EV cargo packaging. They are also integral membrane glycoproteins and as such are processed through the secretory pathway. However, in contrast to PD-L1, CD9 incorporation into EVs was not altered by IPAG, and CD63 incorporation into EVs was enriched with IPAG. These data indicate a selective targeting of EV-associated proteins by a Sigma1 modulator.

Separation of the EVs by iodixanol density gradient fractionation revealed PD-L1 co-isolated into fractions containing CD9, CD81, and CD63, all of which are canonical markers of exosomal EVs (Figure 5). This suggested that some of the Sigma1 inhibitor blocked evPD-L1 that was exosomal.

### Pharmacological inhibition of Sigma1 results in the production of EVs with significantly diminished potency in an assay of T cell inactivation

**Figure 6.**
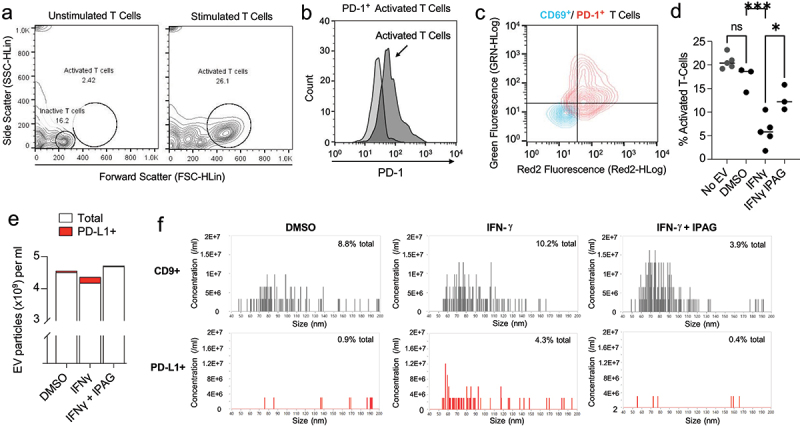
Pharmacological inhibition of Sigma1 reduces the potency of evPD-L1 in an assay of IFN-γ mediated T cell inactivation. (a) T cells isolated from normal human blood were either left unstimulated or activated by CD3/CD28 cross linking for 48–72 hours with the latter then distributed equally into replicate cultures and incubated for 4 hours in the presence or absence of EV preparations from indicated treatments. Activated T cells are larger (> FSC) and more granular (>ssc) than unstimulated T cells by flow cytometry. (b) Quantification of PD-1 on the surface of unstimulated (light gray) versus activated T (dark gray) cells. (c) The activated, isolated T cell populations used in the EV co-culture assay expressed CD69 and PD-1. The flow cytometry profile of unstained cells is shown in blue and double-stained cells in red. (d) EV mediated T cell inactivation assay. PBMCs expressing PD-1 (2,500 cells/well) were co-cultured for 4 hours with 1 × 10^10^ conditioned EVs collected from PC3 cells treated with DMSO, 10 ng/mL IFN-γ, and 10 ng/mL IFN-γ + 10 µmol/L IPAG for 24 hours. An equal number of PC3-derived EVs isolated from the indicated treatment conditions were added to each well of the T cell inactivation assay. Data represent 3 to 5 independent determinations and error bars represent SEM. *p < .05, **p < .01, ***p < .001, ns = no significance. (e) EV particle numbers and PD-L1 status were determined by NanoFCM. The relative number of PD-L1+ EVs particles indicated in red. DMSO (4.23 × 10^7^ PD-L1^+^ EVs of 4.51 × 10^9^ total EVs), IFN-γ (1.81 × 10^8^ PD-L1^+^ EVs of 4.18 × 10^9^ total EVs), IFN-γ + IPAG (1.81 × 10^7^ PD-L1^+^ EVs of 4.69 × 10^9^ total EVs). (f) Representative nano-fcm analysis of PD-L1 bearing EVs from PC3 cells treated with DMSO, 10 ng/mL IFN-γ, and 10 ng/mL IFN-γ + 10 µmol/L IPAG. CD9 (canonical EV marker) was used to identify number of exosome EV particles.

The data above demonstrate that IPAG can decrease the number of evPD-L1 available for the inactivation of T cells entering the TME. Here, we hypothesized that the capacity of EVs produced during IPAG treatment may also have diminished potency in blocking T cell inactivation *in vitro*, as expected by their reduced PD-L1 content. We incubated activated PD-1-positive normal human T cells with EVs prepared from PC3 prostate cancer cells. We compared equal numbers of EVs from untreated PC3 cells with those from PC3 cultured with or without IFN-γ plus or minus the Sigma1 inhibitor IPAG. Cultures of T cells isolated from normal human blood were activated by CD3/CD28 cross linking for 48–72 hours, then distributed equally into replicate cultures and incubated for 4 hours in the presence or absence of the various EV preparations (Figure 6). The cells were then analyzed by flow cytometry and gated for activated T cells, which are delineated by the circular gate in Figure 6a. Activated cells consisted of over 80% CD4 T cells and 10% CD8 T cells with minimal double positives and cells negative for both antigens with these ratios being unchanged by exposure to the EVs (data not shown). Unstimulated cultures generally contained less than 20% of the cells gating as activated and expressing PD-1. In contrast, the majority of the T cells in stimulated cultures exhibited a distinct elevated SSC/FSC scatter and expressed PD-1 (Figure 6a,b). The majority of the activated T cells also expressed the activation marker CD69 in addition to PD-1 (Figure 6c). The inclusion of EVs from untreated PC3 cells resulted in a moderate reduction in the recovery of activated T cells, which was further reduced when the EVs were obtained from IFN-γ-treated PC3 cells. The inhibitory effect on T cell activation was significantly attenuated in the presence of EVs from PC3 cells treated with both IFN-γ and IPAG compared to EVs from cells treated with IFN-γ alone (Figure 6d). The magnitude of the effect on T cell activation by EVs obtained from PC3 cells treated with IFN-γ was consistent with the nanoFCM profiling where the percentage of PD-L1 positive EVs is in the range of 4% of total EVs compared to less than 1% in control (DMSO) and IFN-γ and IPAG combination treatments (Figure 6e). Considering the large number of EVs used in this assay, with relatively few evPD-L1, it is possible that other factors, unaffected by Sigma1 inhibitors, may be contributing to the immune modulatory effects, and this may explain why the IFN-γ + IPAG treatment condition does not completely abrogate the T cell inhibition effect of the EVs.

### Sigma1 targeting selectively sorts proteins into EVs

**Figure 7.**
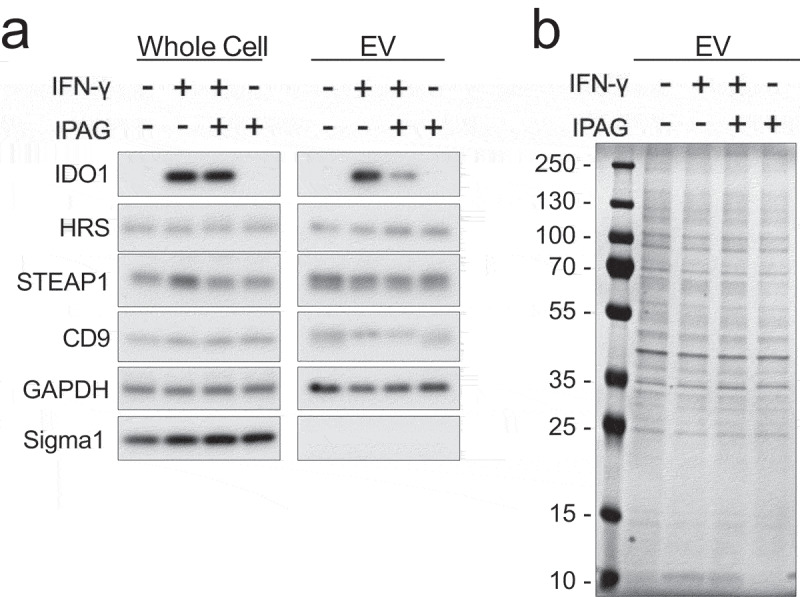
Sigma1 inhibition prevents incorporation of other IFN-γ/STAT1 induced immune suppressive factors into EVs. PC3 cells were treated for 16 hours with DMSO, 10 ng/mL IFN-γ, 10 ng/mL IFN-γ and 10 µmol/L IPAG, or 10 µmol/L IPAG. (a) Immunoblot of PC3 whole-cell protein extracts and lysates of EVs from PC3 cells. 1 × 10^9^ EVs were loaded per lane. Indoleamine 2,3-dioxygenase 1 (IDO), STEAP1, EV markers HRS and CD9, GAPDH, and intracellular integral membrane protein Sigma1. (b) Coomassie stain of SDS-PAGE resolved EV proteins.

Whereas PD-L1 cell surface levels and incorporation into EVs were decreased by IPAG, other transmembrane glycoproteins such as CD9 and CD63 were not excluded from EVs, with the former remaining unchanged between conditions and the latter increasing in EVs (Figure 5). It remains unclear why the relative level of CD63 was increased in EVs. One recently reported explanation for this phenomenon is that inhibited endocytosis can induce a selective increase in vesicular secretion of CD63.^55^ Another integral membrane protein called STEAP1 (six-transmembrane epithelial antigen of the prostate 1), a cell surface antigen for therapeutic targeting in prostate cancer, which has been reported in some PC3 cell lines and PC3 derived EVs,^56,57^ did not change with Sigma1 inhibitor treatment (Figure 7). This is evidence of another EV-associated cell surface protein that is synthesized, processed, matured, and transported through the ER but not excluded from EVs by Sigma1 targeting. This is potentially useful information for future clinical studies as STEAP1 targeting bispecific antibody therapeutics and chimeric antigen receptor T cell (CAR-T) immune therapies are emerging as novel approaches to treating advanced prostate cancers^58,59^ as well as potential diagnostic and prognostic biomarker strategies^60^.

Sigma1 inhibition suppressed the packaging of other adaptive immune resistance factors into EVs. Indoleamine 2,3-dioxygenase 1 (IDO) is induced by IFN-γ^61,62^ and packaged into EVs^62^. Interestingly, the Sigma1 inhibitors did not block intracellular levels of IFN-γ induced IDO; however, Sigma1 inhibition prevented the packaging of IFN-γ induced IDO into EVs (Figure 7). This is important as IDO may contribute to the resistance to immune checkpoint inhibitors.^61^

We also found that Sigma1 inhibition prevented the incorporation of epidermal growth factor receptor (EGFR) into EVs, and in this case, EGFR protein levels did not change, whereas packaging into EVs decreased (Supplemental Figure 2). Altogether, these data suggest that pharmacological targeting of Sigma1 can selectively alter the sorting of proteins into EVs independent of cellular protein levels. Thus, Sigma1 targeting can selectively regulate protein transport through the ER-associated secretory pathway.

## Discussion

evPD-L1 has been reported to suppress anti-tumor immunity within the TME and at distant sites.^19,20^ In this study, we examined the mechanisms by which pharmacological inhibition of Sigma1 can interfere with the IFN-γ/STAT1 axis induced cell surface expression and incorporation of PD-L1 into EVs, two locations where PD-L1 can block antitumor immune responses. A prototypical small molecule Sigma1 inhibitor, IPAG, blocked IFN-γ from upregulating PD-L1 at a post-translational step downstream of STAT1 by preventing complete N-linked glycosylation required for maturation and trafficking of functional PD-L1 to the cell surface. In multiple cancer cell lines, pharmacological inhibition of Sigma1 resulted in PD-L1 retention in the ER and cis-Golgi, as evidenced by Endo H sensitivity.

In this study, we discovered that pharmacological inhibition of Sigma1 prevented PD-L1 packaging into cancer cell-derived EVs. Of note, cancer cells producing EVs devoid of PD-L1 were recently reported to facilitate tumor growth inhibition *in vivo* even when the tumors were able to express cell-surface PD-L1.^20^ Taken together, these data support the further study of Sigma1 inhibitors in modulating immune cell infiltration in *in vivo* models of cancer.

Several standard of care cancer treatments have been reported to cause a significant increase in tumor-derived EV production and alter EV content to promote tumor survival, metastasis, immune evasion, and resistance to therapy.^63–65^ Here, we evaluated not only the quantity of EV particles produced by Sigma1 inhibitor treated cancer cells but also the relative potency of these EVs (Figures 4 and 6). In our experiments, EV particle numbers did not significantly increase in response to Sigma1 inhibitor treatment (Figure 4b). To test the potency of these particles, we used equal numbers of EVs in our T cell inactivation assay (Figure 6b). Under these conditions, the EVs produced by IPAG co-treated cells were significantly less potent in their ability to inactivate T cells (Figure 6a,b). The implications of these data are that pharmacological inhibition of Sigma1 may be used not only to prevent cancer cells from inactivating T cells in direct contact but also to prevent the immunosuppressive actions of EVs in the TME.

A major challenge is to better understand the mechanisms and cellular factors that promote immune resistance in the TME and how to sensitize immunologically unresponsive so-called “cold” tumors to anti-tumor immune responses.^66–69^ The ER plays a central role in regulating the production, processing, and activity of secreted and cell surface proteins as well as the content and production of EVs,^70^ and emerging evidence suggests that targeting the ER can modulate tumor immunity.^71–73^ Here, we show that Sigma1 targeting can regulate the production and content of immune modulatory EVs.

The implication of our data is that T cells coming into the vicinity of cancer cells producing such EVs would be rapidly killed, to the point that the tumor tissues would appear immunologically “cold” even if cancer antigen-reactive T cells were present in the patient’s immune repertoire. As EVs can carry tumor antigens, this may also prevent such antigens from being recognized by T cells in the presence of intact EVs. This raises the question of whether treatment of cancer cells such that their EVs are not cytotoxic could rescue the ability to use these EVs as a tumor antigen source. Our work establishes this question as a rationale for future studies.

Also, our data suggests that Sigma1 targeting regulates the production and packaging of other immune modulatory factors, in addition to PD-L1. Future studies should involve the identification and characterization of immune response and tumor metabolism regulating EV content (protein, lipids, metabolites, nucleotides). Sigma1 targeting impacts cancer cell metabolism^74^ which may impact the tumor immune microenvironment as well.

We have shown that some Sigma1 selective small molecule compounds that have traditionally been categorized as antagonists trigger the unfolded protein response and autophagy and inhibit the growth and proliferation of cancer cells.^39^ In contrast, other putative antagonists of Sigma1, including BD1063, NE100, and BD1047, do not induce these effects or do so only modestly.^38,39^ The classification of pharmacological modulators of Sigma1 as putative agonists and antagonists remains poorly understood (reviewed in^39^). However, we and others have demonstrated that some compounds, which we refer to as Sigma1 inhibitors, phenocopy key effects of shRNA-mediated knockdown and knockout of Sigma1.^38–40,42^ IPAG fits this category of Sigma1 inhibitor compounds, whereas BD1063 and NE100 do not.^38–40,44^ Importantly, although IPAG induces UPR and autophagy, these properties alone may not explain its evPD-L1 suppressive effects. Indeed, we previously showed that thapsigargin and tunicamycin, which both trigger ER stress manifested as UPR, did not trigger degradation of PD-L1 and did not decrease PD-L1 levels.^40^ Altogether, these data suggest a novel and distinct mechanism of action of Sigma1 inhibitors, in which UPR and/or autophagy may be required but are not sufficient to suppress the production of evPD-L1.

EVs alter signaling within the TME in multiple ways, beyond modulation of anti-tumor immunity. Cancer cell EVs can promote the growth, transformation, and survival of originating and neighboring cells with the tumor by way of autocrine and paracrine transfer of oncogenic proteins, mRNAs, miRNAs, and other metabolites and signaling molecules.^26–28^ Cancer cells can also promote their own growth, survival, and dissemination throughout the body by secreting EVs that manipulate stromal and vascular tissue within the TME and at distant metastatic sites.^26–28^ Cancer cell-derived EVs also have been shown to modulate the immune response through enhanced production of immune-suppressing cytokines and the induction of regulatory T cells within the TME.^75^ Therefore, Sigma1 inhibitor-mediated decrease in cancer cell-derived EVs would likely alter the TME in multiple ways; beyond just decreasing the production of immunosuppressive evPD-L1, it may also decrease EV-mediated pro-tumorigenic paracrine signaling and TME remodeling.

It is also important to highlight that pharmacological targeting of Sigma1 may be used to selectively alter the sorting of proteins into EVs. For example, whereas PD-L1 cell surface levels and incorporation into EVs were decreased by IPAG, other transmembrane glycoproteins such as STEAP1, CD9, and CD63 were not excluded from EVs (Figures 5, 7). A few published studies have shown that small molecules specifically targeting their cognate receptors can be used to alter their packaging into EVs.^76–78^ These differ from what we are showing in that these previous studies directly targeted cell surface receptors to prevent their packaging into EVs, while we are targeting an ER scaffolding or chaperone protein that supports the maturation and trafficking of multiple proteins through the ER-associated secretory pathway and their packaging in EVs. As an example, we also found that Sigma1 inhibition prevented the incorporation of EGFR into EVs, and in this case, EGFR protein levels did not change, whereas packaging into EVs decreased (Supplemental Figure 2). This is further evidence that Sigma1 inhibition can regulate components of the endolysosomal sorting machinery to control protein packaging into EVs. Thus, Sigma1 modulation has a broader impact on content and potential use as Sigma1 is expressed in a broad range of tissues, and it is an approach that is not dependent on a single receptor. In another example of small molecule mediated EV cargo sorting, the treatment of promyelocytic leukemia cells with all-*trans* retinoic acid has been reported to increase the production and secretion of EVs and increase packaging of IL-8, VEGF, tissue factor, and mRNA of multiple angiogenesis related transcripts into EVs.^79^ This too differs from Sigma1 mediated sorting, as this describes retinoid-induced transcriptomic changes in target cells, which causes the cell to differentiate into a new cell type with a distinctly altered secretory profile, and not by physically sorting proteins within the secretory pathway.

In conclusion, the data presented here illustrate a novel pharmacological mechanism to promote antitumor immunity using a selective small-molecule modulator of Sigma1. By suppressing both cell surface and evPD-L1, Sigma1 modulators could inhibit adaptive immune resistance by cancer cells, allowing the immune system to be more effective at targeting cancer cells. Furthermore, by altering the content of cancer cell EVs, Sigma1 modulators could prove to be an effective mechanism for preventing pro-tumorigenic remodeling of the TME. These data support the evaluation of Sigma1 small molecule modulators in *in vivo* models of cancer to determine how they reshape the relationship between the immune system and the TME.

## Supplementary Material

Supplemental Material

## Data Availability

The authors confirm that the data supporting the findings of this study are available within the article [and/or] its supplementary materials.
