# Supplementary material for: Sigma1 inhibitor suppression of adaptive immune resistance mechanisms mediated by cancer cell derived extracellular vesicles
Source: Cancer Biol Ther. 2025 Jan 26;26(1):2455722. doi: 10.1080/15384047.2025.2455722 (PMC11776462; doi:10.1080/15384047.2025.2455722)

# A

Hydrostatic Filtration Dialysis (HFD)


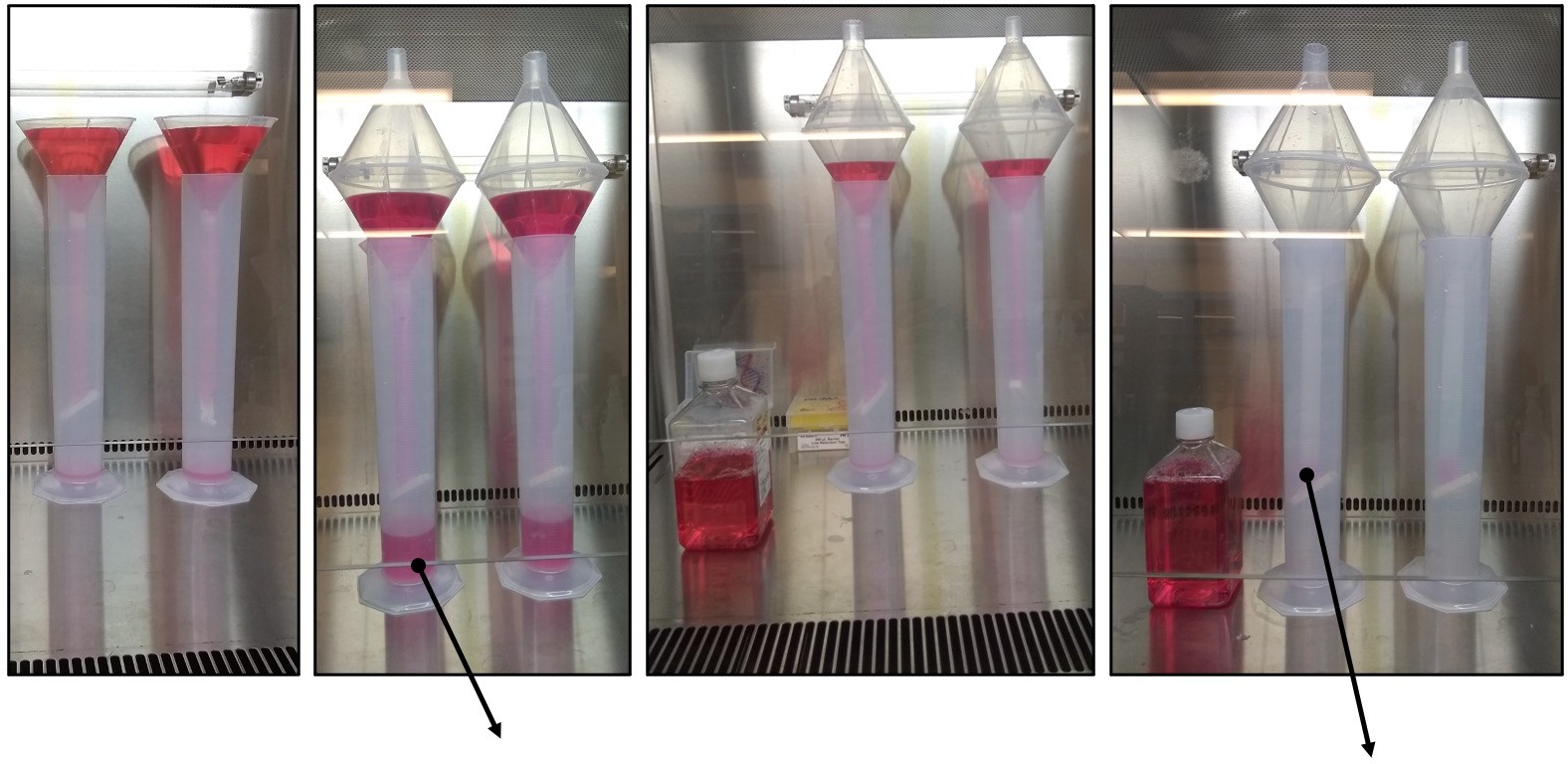
B C


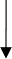
 rav-rmax


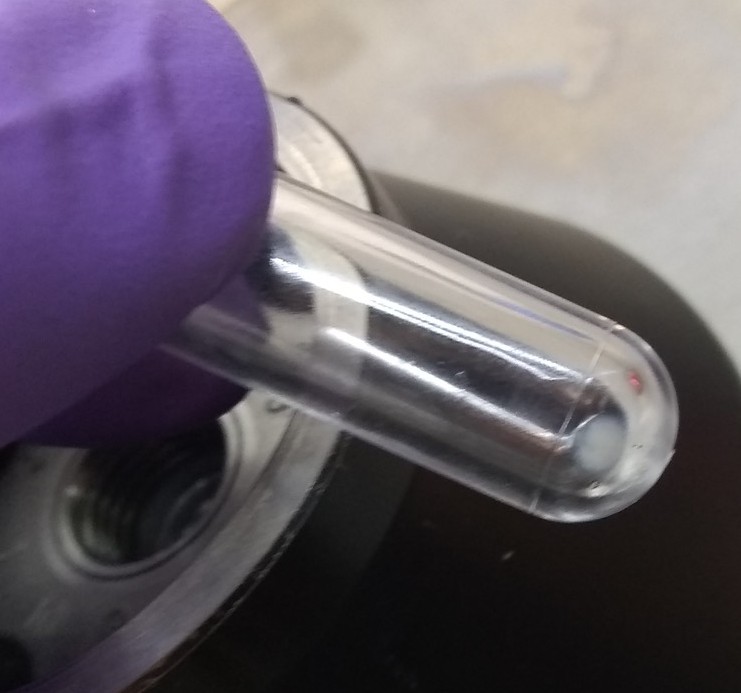


**HFDa**

14,000 rpm = 18,000-20,000g 35’ 4℃


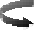

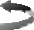


P20 (crude)


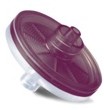
2 mL PBS0.1µm


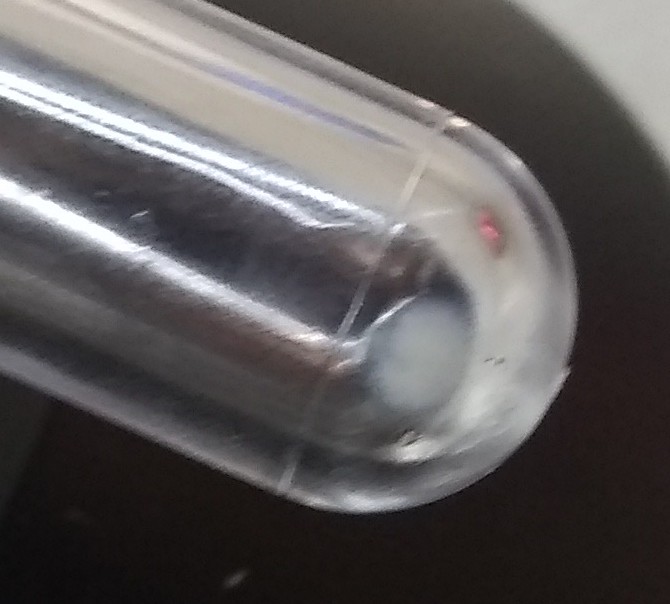


rmin-rmax

25,000 rpm = 17,000-27,000g 45’ 4℃


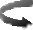

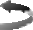


Flow through : HFDb

Retentate: HFDa

SN20 discarded

0.2 mL PBS0.1µm

P20 (washed)

**Supplemental Figure 1. Procedure for isolation of extracellular vesicles (EVs) from cell culture medium.** To isolate EVs from > 1 liter volumes of cell culture medium, we precleared precleared the medium by centrifugation to remove dead cells and debris (conditioned medium, CM), and subsequently we performed hydrostatic filtration dialysis (HFD) to initially concentrate EVs, followed by high speed/ultracentrifugation. **(A)** Illustration of HFD set-up. Filtration membrane were fixed at onto funnels and assembled inside graduated cylinders. Conditioned medium poured into cylinder flowed through by gravity. The flow through fraction, HFDb, was not used for experiments. EVs were isolated from the retentate, HFDa, by ultracentrifugation. **(B)** Ultracentrifugation work flow of HFDa retentate fraction. HFDa fraction was centrifuged at 18 - 20,000 x g for 35’ to isolate initial, crude “P20” (indicating EV pellet collected by centrifugation at 20,000 x g) pellet, then 2 mL of PBS filtered through 0.1 micron (um) filter was added to the crude P20 pellet and subsequently centrifuged 17,000 – 27,000 x g for 45 minutes. The supernatant (SN20) was discarded, and the final, washed P20 EV pellet was used for experiments. **(C)** Photo of resulting EV-P20 pellet.

A **Whole Cell EV**


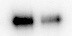

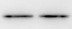

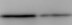

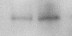

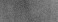

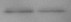

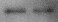


DMSO

IPAG

DMSO

IPAG

- 1. **EV Silver Stain** C

**DMSO IPAG**


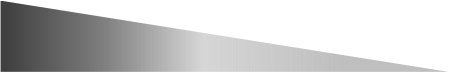


Density Gradient Fractions


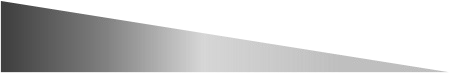


Density Gradient Fractions

EGFR


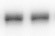

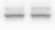

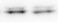

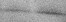

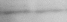

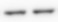

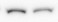


CD9 CD81

Syntenin

HRS TSG101

Calnexin

EGFR

DMSO

IPAG

HRS CD81


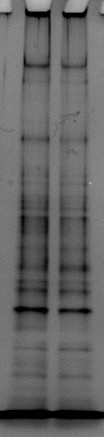


Syntenin

CD9

CD63

10 9 8 7 6 5 4 3 2 1

10 9 8 7 6 5 4 3 2 1

**Supplemental Figure 2. Sigma1 inhibitor prevents epidermal growth factor receptor (EGFR) incorporation into extracellular vesicles (EV) without decreasing total cellular EGFR protein levels**. MDA-MB-231 cells were treated for 24 hours with DMSO or 10 µmol/L IPAG. **(A)** Immunoblot of whole cell protein extracts and EVs from MDA-MB-231 cells. 1 x 109 EVs were loaded per lane. **(B)** Silver stain SDS-PAGE gel of EVs from DMSO and IPAG-treated MDA-MB-231 cells. 1 x 109 EVs were loaded per lane. **(C)** Immunoblots of EVs separated on an iodixanol density gradient showing EGFR colocalizing in fractions with canonical EV markers HRS, CD81, Syntenin, CD9, and CD63. Fraction 10 (most dense) to 1 (least dense) fractions.


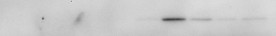

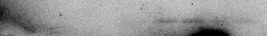

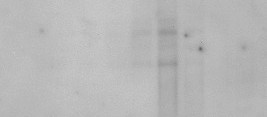

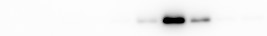

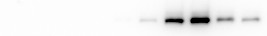

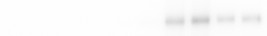

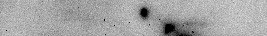

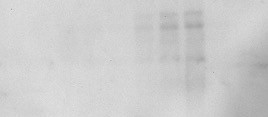

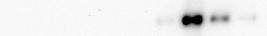

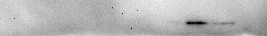

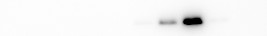

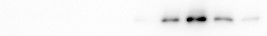

Supplement: Supplemental Material [file KCBT_A_2455722_SM1466.docx]
